# Dataset of lignocellulosic residue valorization of cropland activities to produce activated carbon

**DOI:** 10.1016/j.dib.2025.112303

**Published:** 2025-11-20

**Authors:** Leonel E. Amabilis-Sosa, Alejandro David Ortiz-Marin, Kimberly Mendivil-García, Jorge Chavarria, Oscar Joaquín Solís-Marcial, Raudel Medina-Leaños, Adriana Roé-Sosa

**Affiliations:** a División de Estudios de Posgrado e Investigación, Tecnológico Nacional de México/IT de Culiacán, *Av*. Juan de Dios Batiz No 310, Culiacán, 80220, Sinaloa, Mexico; b Secretaría de Ciencia, Humanidades, Tecnología e Innovación, *Av*. Insurgentes Sur 1582, Benito Juárez, 03940, Ciudad de México, Mexico; c Tecnológico de Monterrey, Escuela de Ingenieria y Ciencias, Culiacan 80100, Sinaloa, Mexico; d Universidad Autónoma Metropolitana, Unidad Azcapotzalco, Departamento de Energía, *Av* San Pablo Xalpa 180, Azcapotzalco, 02128, Ciudad de México, Mexico; e Instituto Politécnico Nacional, Unidad Profesional Interdisciplinaria de Ingeniería, Zacatecas, 98160, Zacatecas, Mexico; f Universidad Tecnológica de Culiacán, Coordinación de Tecnología Ambiental, Culiacián, 80014, Sinaloa, Mexico

**Keywords:** Agricultural residue, Lignocellulosic residue, Valorization, Activated carbon, Environmental applications

## Abstract

This dataset compiles experimental and geospatial information on the valorization of agricultural lignocellulosic residues from Zacatecas, Mexico, for potential conversion into activated carbon. Data acquisition involved a combination of field sampling, laboratory analysis, and Geographic Information System (GIS) processing. Four primary crops were selected, corn, oats, agave, and garlic due to their significant residue generation. Residues were collected and characterized for their cellulose, hemicellulose, lignin, ash, and nutrient content following standardized protocols, including the Neutral Detergent Fiber (NDF) method and sequential Van Soest methodology. Nutrient analysis was performed using the brucine sulfate method for nitrogen and the ascorbic acid technique for phosphates. All analyses were conducted in triplicate to ensure reproducibility, and the coefficient of variation was calculated to identify possible outliers.

Complementary to experimental work, spatial data were obtained using QGIS software and official geospatial datasets. These included digital elevation models, land use maps, and administrative boundaries. Layers were integrated to generate land use and vegetation distribution maps, supported by zonal statistics and reclassification tools. Production and yield statistics for 2023 were extracted from the Servicio de Información Agroalimentaria y Pesquera (SIAP) database, allowing for the calculation of residue generation per crop through residue-to-product ratios. The dataset integrates thematic and spatial data across multiple layers—municipalities, land use, and hydrological regions—allowing a comprehensive territorial analysis of biomass availability.

The repository includes raw and processed data in formats compatible with Excel and QGIS. It is organized into directories containing land-use shapefiles, municipality coordinates, crop production tables, and physicochemical characterization files. Tables provide detailed information on harvested area, production volume, yields, estimated residue generation, lignocellulosic composition, nutrient content, and conversion yields to activated carbon.

This dataset has the potential for reuse in diverse contexts. It can be applied in comparative studies of biomass valorization across regions, extended to evaluate other agricultural systems using similar GIS and residue quantification methods, or integrated into models of circular economy (CE) strategies. The structured information is also suitable for environmental planning, crop residue management assessments, and as a reference for laboratory-scale experiments on biomass conversion technologies. Furthermore, its reproducibility and spatially explicit design support applications in academic research, sustainable resource management, and decision-making in agricultural and environmental policy.

This study more clearly addresses the existing research gap in the valorization of lignocellulosic agricultural residues by integrating, for the first time, an experimental and geospatial approach to quantify, characterize, and map the territorial availability of biomass in Zacatecas, Mexico. This methodological integration represents a specific and relevant contribution to the scientific literature, providing a solid foundation for the sustainable planning of agricultural residue utilization and the development of circular economy strategies at the regional or national levels.

Specifications Table

**Table:** 

Subject	Engineering & Materials science
Specific subject area	Activated carbon from agricultural residues effectively supports of transition metals to enhancement catalytic capability.
Type of data	Tables, charts, image, processed data, raw, and analysed data.
Data collection	The dataset was constructed using experimental data and data extracted from four agricultural field classes from a geographic information system. The experimental results include cellulose, hemicellulose, and lignin, obtained from representative samples of agricultural residues (i.e., oats, corn, agave, and garlic). The four agricultural field classes are A, B, C, and D. The lignocellulosic residues were obtained from the land use study in Mexico using Qgis geographic information system software. The physicochemical analysis was performed using the standardized Neutral Detergent Fiber (NDF) method, which allows measuring the cell wall fraction in lignocellulosic materials, including cellulose, hemicellulose, and lignin. The experimental data were analysed in triplicate, and the coefficient of variation was calculated to ensure reproducibility and to identify potential outliers.
Data source location	Laboratory of metallurgy of the Interdisciplinary Professional Unit of Engineering Zacatecas Campus (UPIIZ-IPN), Zacatecas, México. (22.784154404297514, −102.6175155). Here, the data collected from agricultural fields and extracted from the Geographical Information System were processed. Calera: 102°54′28.80″ W 102°36′10.80″ W, 22°50′08.16″ N 23°07′18.84″ N Fresnillo: 103°31′26.40″ W 102°29′06.00″ W, 22°51′10.80″ N 23°35′47.04″ N Guadalupe: 102°39′25.20″ W 102°11′49.20″ W, 22°32′23.28″ N 23°01′29.64″ N Morelos: 102°47′45.60″ W 102°34′08.40″ W, 22°46′36.48″ N 22°56′02.40″ N Zacatecas: 102°50′60.00″ W 102°32′20.40″ W, 22°37′01.92″ N 22°50′29.40″ N
Data accessibility	Repository name: Mendeley Data respository Data for publication/ Data identification number: 10.17632/79pn92jpr2.3 Direct URL to data: https://data.mendeley.com/datasets/79pn92jpr2/3
Related research article	None

## 1 Value of the Data

• This dataset addresses a critical limitation in existing repositories by integrating experimental physicochemical characterization with geospatial analysis of lignocellulosic agricultural residues—an approach rarely found in publicly accessible biomass datasets. Whereas previous studies have concentrated mainly on laboratory-scale material characterization or isolated regional residue inventories, this dataset provides a comprehensive and spatially explicit framework that connects the compositional properties of residues with their territorial distribution and valorization potential. This integrated perspective enables a more systematic understanding of how agricultural residues can be sustainably managed and converted into value-added materials.
• It provides detailed insights into the production and functionalization of activated carbon as a low-cost, sustainable alternative to conventional adsorbents and catalysts in advanced oxidation processes (AOPs). This technological contribution expands the library of materials available for environmental purification and catalytic applications. The dataset can be used to conduct comparative studies in different regions. Similar geoprocessing and GIS methodologies can be applied to identify areas with high potential for the valorization of agricultural residues.
• The inclusion of geospatial layers allows for comparative studies across different regions, offering a replicable methodological framework that employs GIS-based residue mapping and geoprocessing tools to identify areas with high potential for agricultural residue valorization.
• The dataset facilitates evidence-based decision-making in sustainable development projects, circular economy strategies, and the decentralized production of activated carbon for environmental applications.
• Potential users include environmental engineers, agricultural planners, materials scientists, policy analysts, and circular economy strategists interested in biomass valorization, resource efficiency, and sustainable material design.

## 2 Background

The study of agricultural residues has gained increasing relevance in recent years, closely aligned with the United Nations Sustainable Development Goals (SDGs) on responsible production, climate action, and innovation [1]. Their inadequate management is recognized as a significant source of greenhouse gas emissions, intensifying global warming [2]. Therefore, residue recovery and valorization strategies are crucial to strengthening circular economy frameworks and developing sustainable materials [3] in developing countries.

Mexico, with its high agricultural productivity, offers significant opportunities for residue valorization [4]. In Zacatecas (North-Central Region), lignocellulosic residues from corn, oats, agave, and garlic show favorable physicochemical characteristics for conversion into activated carbon, a key material in environmental remediation [5]. In 2021, the Agri-Food and Fisheries Information Service (SIAP) reported 190,872.02 tons of forage corn, 390,813.43 tons of grain corn, 1333,325.73 tons of forage oats, 4090.00 tons of grain oats, 4155.00 tons of agave, and 44,657.15 tons of garlic [4]. These figures highlight the potential to develop value chains that promote regional bioeconomy and alternative farmer incomes [[8], [9]].

The integration of physicochemical data with geospatial and land-use layers provides an added scientific value, as it enables a spatially explicit and quantitative assessment of how residue management practices affect soil quality, biomass potential, and environmental risks in specifically sites. This interdisciplinary approach allows identifying priority zones (“hotspots”) for residue valorization based on both material quality and territorial context. Moreover, it strengthens data interoperability and alignment with the circular economy, contributing to open and reproducible science. The combination of physicochemical and spatial data situates this dataset within current global discussions on biomass valorization, sustainable resource mapping, and circular economy data infrastructures, supporting evidence-based policy and sustainable development planning [[7], [8], [9]].

## 3 Data Description

The dataset is organized within the primary and secundary data directory, which contains both primary physicochemical data and secondary geospatial information. This structure enables the integration of laboratory analyses with spatial layers to support territorial and environmental assessments related to biomass valorization.

Primary data correspond to the experimental characterization of agricultural residues generated in the state of Zacatecas, Mexico. The samples analyzed include corn stover, oat straw, agave bagasse, and garlic peel, which were selected for their abundance and lignocellulosic composition.

Each residue sample was characterized following standardized laboratory protocols. Table 1 provides a summary of the raw and processed datasets, detailing the variables measured and their corresponding units and categories.

**Table 1:** Structure and variables of the physicochemical dataset.

Variable name	Description	Unit / Data type	Category
Sample_ID	Unique identifier for each residue sample	Text	Metadata
Residue_type	Type of agricultural residue (corn, oats, agave, garlic)	Text	Physicochemical
Ash_content	Inorganic residue after combustion	%	Physicochemical
Fixed_carbon	Carbon fraction remaining after volatile release	%	Physicochemical
C, H, O, N,P	Physicochemical composition (lignin, cellulose, hemucellulose and nutrient content)	%	Physicochemical

Also, in Table 2, the raw dataset (Table_corps_waste) compiles quantitative agricultural indicators —planted, harvested, and damaged areas; production; yield; efficiency; and damage percentage— used to assess crop performance and residue generation potential. The processed dataset (Table_crops_waste) integrates analytical results derived from the raw data, including yield classification, estimated waste quantities, and potential reuse pathways. These processed variables support decision-making for residue valorization and circular economy strategies at the regional level. Both datasets are provided in tabular format (.XLS).

**Table 2:** Structure and description of the dataset variables.

File	Data name	Units	Data type
Table_corps_waste	Planted, harvested, damaged, production and yield, efficiency, damaged	ha, ha, ha, ton, ton/ha, % and % respectively.	Raw data
Table_crops_waste	Yield classification	Text	Processed data
Table_crops_waste	Stimated waste	Ton	Processed data
Table_crops_waste	Waste characteristics	Text	Processed data
Table_crops_waste	Possible reuse	Text	Processed data

**Fig. 1:**
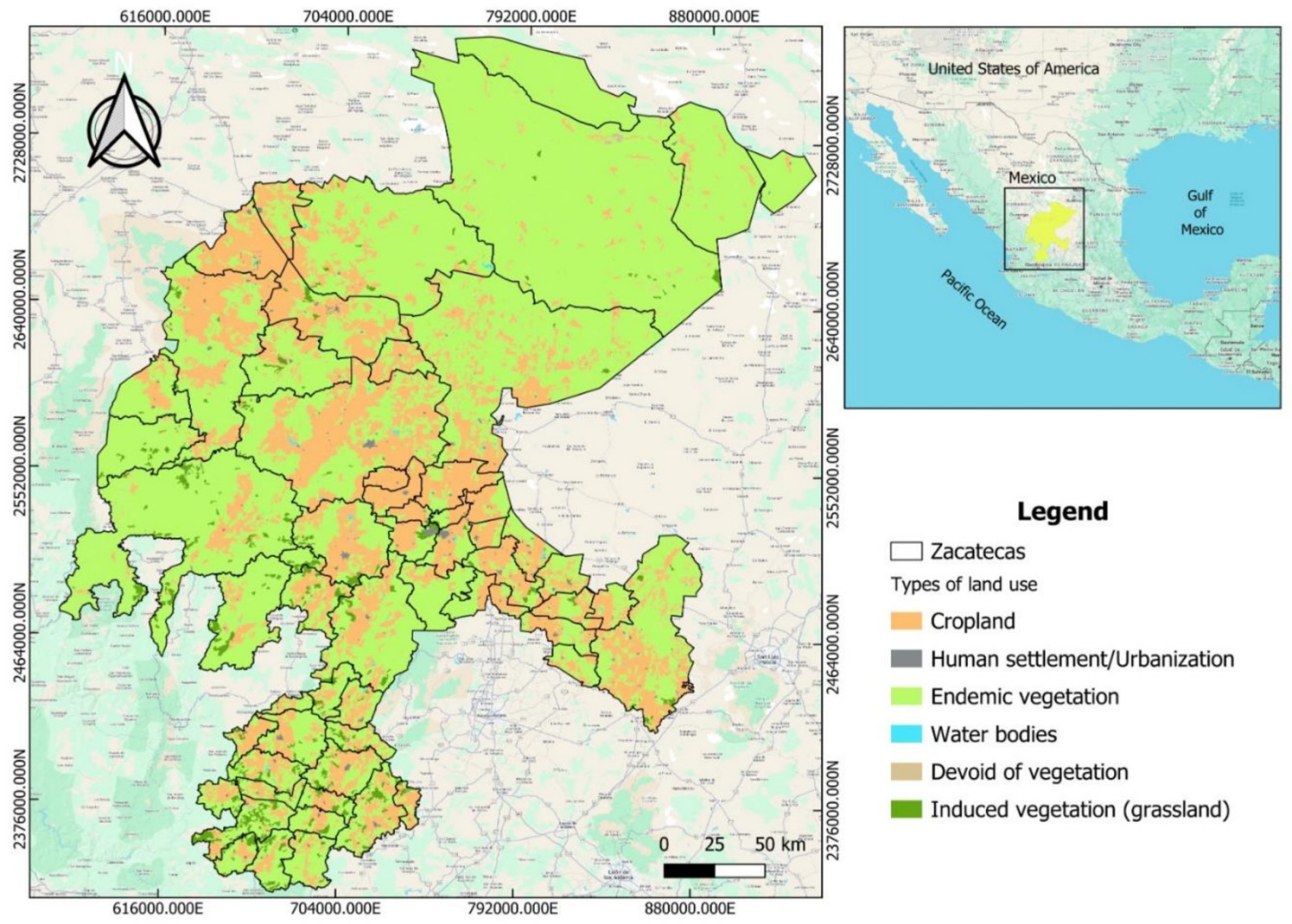
Thematic map of the state of Zacatecas, Mexico, showing the distribution of land use and vegetation cover.

Secondary data (Table 3) consist of the land use, municipalities, hydrological regions, and Zacatecas datasets, which together integrate spatial and thematic information relevant to territorial analysis. These datasets were obtained from publicly available sources, primarily the Instituto Nacional de Estadística y Geografía (INEGI, Series II, 2025) and other official GIS repositories. The data are organized into three main components: the municipalities layer, which defines local administrative boundaries and official names; the land use layer, which classifies predominant activities and land cover types—agricultural, urban, forestry, livestock, and protected areas—reflecting the region’s productive and ecological diversity; and the hydrological regions layer, which divides the territory into basins and sub-basins, enabling the analysis of water resource dynamics and their influence on land management [6]. These spatial layers serve as geographic references for contextualizing residue sources and identifying potential biomass-generation zones based on geographic coordinates of areas with higher crop activity. By integrating these datasets into a unified spatial framework, the project enables a comprehensive analysis of the interactions among political administration, land cover, and hydrological systems, providing a robust foundation for environmental, agricultural, and urban planning, as well as for sustainable decision-making in the region (Fig. 1).

**Table 3:** Structure and description of the secondary geospatial datasets used in the study.

File / Layer name	Description	Format	Data type
Metadata	Set of vector data from the Land Use and Vegetation map, scale 1:250,000, series II.	.docx	Raw
Data Qgis	Land use and vegetation map for Zacatecas, including categories such as agricultural, pasture, forest, and urban areas.	Shapefile (.shp) / GeoPackage (.gpkg)	Raw
Coordinates by municipality	Coordinates of each area with higher crop activity.	.xls	Raw

In addition to the above, the integration of physicochemical analyses with soil-climate and yield variables contributes significantly to the fulfilment of the Sustainable Development Goals (SDGs), favours the transition to a circular economy, and promotes more efficient and responsible agricultural management. It also helps to establish a framework for information management and analysis in universities and research centres, particularly in the field of statistical applications, using robust databases and the generation of high-quality information.

## 4 Experimental Design, Materials and Methods

The secondary data for this study consist in official geospatial data sets, including raster layers (digital elevation models and land cover maps) and vector layers (administrative boundaries, road networks, and hydrological features) and its metadata were developed following the ISO 19,115 Geographic Information Metadata Standard and are being updated in accordance with the Mexican Technical Standard for the Preparation of Geographic Metadata (NTM) to maintain interoperability and compliance with national data management protocols.

Data processing was conducted using QGIS version 3.32. All spatial data were standardized to a consistent coordinate reference system (WGS 84) and resampled to a uniform spatial resolution (1:250,000) to ensure scale compatibility and analytical consistency across layers. Spatial analyses were performed using QGIS tools such as Raster Calculator, Zonal Statistics, and Reclassify, allowing for the extraction and quantification of relevant territorial attributes.

Based on these datasets, quantitative analyses of crop production were carried out to identify the highest-yielding crops, followed by the estimation of agricultural residue generation from these major crops. The assessment recognized potential biases associated with spatial resolution, regional variability, and conversion coefficients used in residue estimation. To ensure data quality and reproducibility, all intermediate and final datasets were archived in Microsoft Excel (.xlsx) and Geospatial formats, accompanied by metadata that document data sources, processing steps, and validation methods.

The spatial scale for residue quantification was validated by comparing municipal and state production data to ensure representativeness, although potential biases may result from differences in data reporting, temporal coverage, and spatial resolution. This section also includes the lignocellulosic characterization of corn, oats, agave, and garlic residues, providing essential information for assessing their suitability for biomass valorization and circular economy applications. The selection of residue-to-product ratios was justified based on established literature, national agricultural standards, and previous studies conducted by the authors. These ratios were calculated and, when necessary, adapted from peer-reviewed sources to ensure methodological consistency, representativeness, and comparability across the analyzed crops [10].

### 4.1 Map generation

QGIS software was used to create the land use map for the state of Zacatecas, utilizing vector and raster geospatial layers sourced from official sources and verified to ensure their topological accuracy and georeferencing within the WGS 84 system [7]. The land use categories considered included cropland, endemic vegetation, induced vegetation (grasslands), water bodies, human settlements, and areas lacking vegetation. Different color symbologist were applied for each category, facilitating spatial visualization and analysis of territorial distribution. The resulting map clearly identifies the predominance of endemic vegetation and grasslands in the territory, as well as the concentration of agricultural and urbanized areas. It serves as a basis for territorial planning studies, natural resource management, and environmental analysis in the region. Finally, the cartographic product was exported in high-resolution format, ensuring its reproducibility and usefulness for scientific publications.

### 4.2 Data on crops harvested in zacatecas

Open-access data on the different crops in the state of Zacatecas were obtained from the SIAP website (Table 4) [8]. Once the data on crops, species, and total production for the year 2023 were obtained, the estimated residues produced by each crop were calculated, along with the characteristics of these residues and their possible reuse for recovery. This new data obtained from the analysis of each crop was used to generate new columns with processed data, which can be found in the repository (Table 1).

**Table 4:** Data from the total crops harvested, production, yield, estimated residue production, and qualitative characteristics for the valorization of each [10].

Crop	Harvested surface (ha)	Production (ton)	Yield (ton/ha)	Estimated residue (ton)	Residue characteristics	Possible reuse
Agave tequilana	50	4155	83.1	88,418.4	Lignocellulosic fibers, high cellulose content	Bioethanol, composting, activated carbon
Alfalfa	15,787.65	1375,920.51	87.15	0	Miscellaneous plant residue	Composting
Apple	688.5	4963.09	7.21	122.57	Pulp, peels and seeds	Composting, biogas, natural extracts
Avocado	44	338.2	7.69	11.535	Miscellaneous plant residue	Composting
Barley grain	18,323.03	42,246.15	2.31	16,698.99	Dry straw and stalks	Bioenergy, composting, activated carbon
Bean	603,408.27	351,485.15	0.58	325.49	Miscellaneous plant residue	Composting
Broccoli	369	4985.54	13.51	0	Miscellaneous plant residue	Composting and animal feed
Cactus	367	12,411.31	33.82	33.82	Miscellaneous plant residue	Composting
Cactus fruit	11,523.5	100,607.08	8.73	1283.31	Miscellaneous plant residue	Composting
Carrot	2151.8	72,099.98	33.51	3446.84	Miscellaneous plant residue	Composting and animal feed
Cauliflower	323.1	7613.05	23.56	0	Miscellaneous fresh plant residue	Composting and animal feed
Corn grain	174,721.06	390,813.43	2.24	66,599.23	Stems, leaves, lignin and cellulose	Bioethanol, pellets, activated carbon
Cucumber	303.56	19,175.81	63.17	0	Miscellaneous fresh plant residue	Composting and animal feed
Fresh forage oats	103,553.75	1333,325.73	12.88	5535.57	Green biomass with high moisture content	Animal feed, biogas, composting
Garlic	2777.61	44,657.15	16.08	833.28	Stems and husks, sulfur compounds	Composting, antimicrobials, activated carbon
Grape	6855.15	83,262.08	12.15	8233.44	Pulp, peels and seeds	Composting, biogas, natural extracts
Green chilli	36,608.29	424,081.57	11.58	0	Miscellaneous fresh plant residue	Composting and animal feed
Green forage corn	95,847.17	1908,724.02	19.91	5933.18	Stems, leaves, lignin and cellulose	Composting and animal feed
Green forage sorghum	2584	47,697.4	18.46	738.4	Dry straw and stalks	Bioenergy, composting, and activated carbon
Gren Tomato	3141.78	92,639.77	29.49	0	Miscellaneous fresh plant residue	Composting and animal feed
Guava	1667	29,982	17.99	0	Pulp, peels and seeds	Composting, biogas, natural extracts
Lemon	467.2	6242.54	13.36	2658.64	Pulp, peels and seeds	Composting, biogas, natural extracts
Lettuce	3810.63	96,088.28	25.22	0	Miscellaneous fresh plant residue	Composting and animal feed
Mango	28	207.3	7.4	0	Pulp, peels and seeds	Composting, biogas, natural extracts
Melon	10	350	35	0	Pulp, peels and seeds	Composting, biogas, natural extracts
Nut	118	306.5	2.6	31.2	Miscellaneous plant residue	Composting
Oats grain	920	4090	4.45	184	Miscellaneous plant residue	Composting
Onion	5458.93	215,986.3	39.57	0	Miscellaneous fresh plant residue	Composting and animal feed
Orange	1.8	18.25	10.14	0	Pulp, peels and seeds	Composting, biogas, natural extracts
Peach	11,324.15	66,629.3	5.88	2754.78	Pulp, peels and seeds	Composting, biogas, natural extracts
Pear	16	113.8	7.11	78.21	Pulp, peels and seeds	Composting, biogas, natural extracts
Potato	789	34,390.07	43.59	0	Miscellaneous plant residue	Composting
Red Tomato	2422.23	158,970.25	65.63	0	Miscellaneous fresh plant residue	Composting and animal feed
Sorghum grain	6	24.3	4.05	923.4	Stems, leaves, lignin and cellulose	Bioenergy, composting, activated carbon
Strawberry	22.12	272	12.3	0	Miscellaneous plant residue	Composting
Watermelon	10	408.8	40.88	0	Pulp, peels and seeds	Composting, biogas, natural extracts
Wheat grain	8264.16	20,817.02	2.52	5992.36	Dry straw and stalks	Bioenergy, composting, activated carbon
Zucchini	850.35	27,409.44	32.23	0	Miscellaneous fresh plant residue	Composting, biogas, natural extracts

Four representative crops such as corn, oats, agave, and garlic were selected for physicochemical characterization. These crops were prioritized because they represent the highest production volumes and residue generation in the Zacatecas region, based on data from the Agri-Food and Fisheries Information Service (SIAP, 2021–2023). Their selection ensures that the dataset captures the dominant agricultural systems and provides a robust basis for evaluating biomass valorization potential.

The agricultural residue valuation table was developed based on a research and analysis process carried out in several phases. Initially, crops of interest were selected based on their agricultural relevance and the residues generated. After identifying the crops, the residual biomass was estimated using annual production data and residue/product ratio factors, which made it possible to quantify the volume of raw material available. Subsequently, a physicochemical characterization of the residue was carried out to determine its main composition, with a focus on lignocellulose content. Finally, the conversion yields of this residue into activated carbon were investigated, extracting data from laboratory-scale experimental studies. Consolidating all this information into a table allowed the recovery potential of each agricultural residue to be visualized and evaluated (Table 5).

**Table 5:** Crop residue information of valorization and kg of AC per ton residue [10].

Crop of interest	Estimated residue (ton)	Kg AC/ ton residue	residue type	Main Characteristics	Possible Application (valorization)
Agave tequilana	88.4k	5.48k	Lignocellulosic fiber	Lignocellulosic fiber	Biofilm and activated carbon
Oats grain	818	204.5	Straw	Starch and lignocellulosic	Activated carbon and biofuels
Fresh forage oats	5.5k	1.1k	Biomass	Cellulose	Activated carbon
Garlic	833	183.26	Stem and husk	Lignocellulosic	Activated carbon
Corn grain	66.6k	7.9k	Stem and leaves	Lignocellulosic	Pellets and activated carbon

### 4.3 Quantification of lignocellulosic content

To evaluate the cellulose, hemicellulose, and lignin content in the biomass, the Van Soest sequential methodology was applied, which involves three stages of digestion with detergents and acids. Initially, a neutral detergent was used to dissolve the soluble components, leaving a residue called neutral detergent fiber (NDF), which consists of cellulose, hemicellulose, and lignin. This residue was then treated with an acid detergent to remove the hemicellulose, resulting in acid detergent fiber (ADF), composed of cellulose and lignin. Finally, the lignin was separated from the ADF by digestion with concentrated sulfuric acid. This process allowed the percentage of each component of the dry biomass to be calculated using specific formulas that considered the weights of the samples and residues and was complemented by the determination of the ash content by incineration for a complete analysis of the fiber composition (Table 3).

### 4.4 Characterization of nutrient content

For nitrate quantification, the University of Cambridge protocol was followed, which is based on sample extraction with a calcium sulfate solution prepared at 20 °C. Once extracted, nitrogen levels were quantified using the brucine sulfate method, according to standard NMX-AA-079-SCFI-2001, with samples read in a spectrophotometer at a wavelength of 410 nm. In the case of phosphates, the ascorbic acid technique, based on the Olsen method, was used. The principle of this method lies in the reaction of orthophosphate in an acidic medium with ammonium molybdate and potassium antimony tartrate, forming a heteropolyacid that is subsequently reduced by ascorbic acid to produce a blue compound, which can be measured spectrophotometrically to determine the concentration of phosphates in the sample (Table 6).

**Table 6:** Lignocellulosic, ash and nutrient content of the four residues with more possible applications [10].

Crop residue	Ash (%)	Cellulose (%)	Hemicellulose (%)	Lignin (%)	Inorganic compounds (%)	Nitrogen (mg/kg)	Phosphate (mg/kg)
Agave tequila	19.33	30.83	16.5	8	19.33	15	17.5
Oats	7.5	32	25	18	7.5	16	20
Corn	12.67	16.33	34	27	12.67	3.65	42.36
Garlic	10.81	11.5	9.67	26.33	10.81	40.49	130

### 4.5 Suitability index calculation

**Fig. 2:**
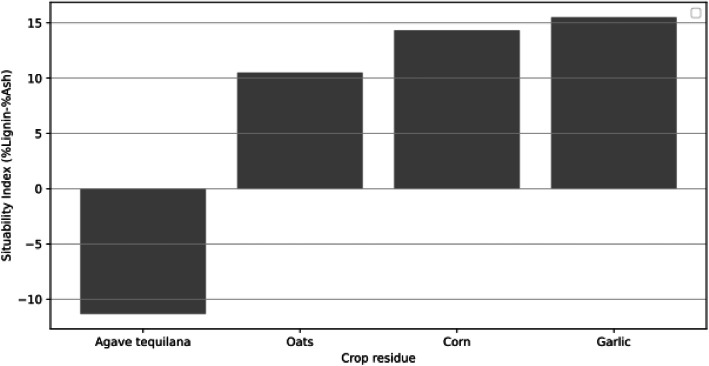
Suitability index of activated carbon production.

The suitability index was calculated by subtracting the ash percentage from the lignin percentage for each type of agricultural residue. Therefore, a positive and high index value indicates greater suitability, as the lignin content significantly exceeds that of ash, suggesting better performance in activated carbon production (Fig. 2).

## Limitations

The dataset provides comprehensive information on the volume of crops processed in the State of Zacatecas and the corresponding annual yields, as well as on the residues generated from these activities and their potential to produce activated carbon. Nevertheless, in many Latin American countries, the generation of agricultural production data is primarily undertaken by governmental agencies. While territorial differentiation is often considered in the development of spatially explicit maps, in many instances the reported values rely on theoretical estimations derived from interpolation methods. Thus, the main limitation lies in the level of resolution and accuracy of the information per unit area. Additionally, laboratory analyses were performed under controlled conditions that ensure internal consistency but may limit reproducibility under different analytical settings. Spatial extrapolation of residue data beyond the Zacatecas region should be interpreted with caution, as variations in agricultural practices, soil properties, and climate conditions may affect residue generation and composition. These limitations also highlight opportunities for future dataset expansion, regional validation, and integration with other open-access repositories to enhance interoperability and broader applicability.

## Ethics Statement

The current work does not involve human subjects, animal experiments, or any data collected from social media platforms.

## CRediT Author Statement

**Alejandro David Ortiz-Marin:** Conceptualization, Investigation, Methodology, Resources, Writing - Original Draft; **Leonel Ernesto Amabilis-Sosa:** Conceptualization, Methodology, Validation, Formal analysis, Writing - Original Draft; **Jorge Chavarria:** Writing - Review & Editing, Formal analysis, Data Curation; **Kimberly Mendivil-García:** Methodology, Software, Investigation; **Oscar Joaquín Solis-Marcial:** Formal analysis, Data Curation, Writing - Review & Editing; **Raudel Medina Leaños:** Methodology, Investigation.

## Data Availability

Mendeley DataData for publication (Original data).
